# Does Margin Call Pressure Affect the M&A Decision of Controlling Shareholders With Equity Pledges: Evidence From Chinese Listed Company

**DOI:** 10.3389/fpsyg.2022.898118

**Published:** 2022-04-28

**Authors:** Xintao Li, Qian Li

**Affiliations:** School of Business and Management, Shanghai International Studies University, Shanghai, China

**Keywords:** margin call pressure, controlling shareholder, equity pledge, mergers and acquisitions, agency theory, G18, G30, G34

## Abstract

The equity pledge has become a universal practice in the world. Taking Chinese listed companies from 2007 to 2020 as a sample, this paper investigates the influence of controlling shareholders’ equity pledges on M&A behavior. It is found that the probability of M&A of listed companies is greater when the controlling shareholder has an equity pledge (or the proportion of equity pledge of controlling shareholder is higher). M&A activities can raise the stock price and play the role of market value management. Controlling shareholders with margin call pressure have stronger motivation to use M&A to increase their share price. Investors seem to be aware of this behavior and underestimate the potential benefits of M&A under this pressure. Propensity score matching (PSM) and difference in difference (DID) were used to test endogeneity and the results were still robust. Overall, our results reveal the impact of equity pledges by controlling shareholders on M&A. Furthermore, margin call pressure, this organizational psychology promotes this possibility.

## Introduction

Equity pledge has been widely concerned by media and financial institutions worldwide and has become a global phenomenon in recent years. Equity pledge occurs when equity is used as collateral for personal loans (Chan et al., 2018). According to a survey in the United States, 982 directors or senior managers publicized that they had a high share pledge in the announcement from 2006 to 2009 (Larcker and Tayan, 2010). Press Trust of India (PTI) reported that more than 20% of insiders of listed companies in India pledged part of their shares, and the pledged market value in 2013 was US$ 25.8 billion (Chan et al., 2018). In China, the occurrence and number of equity pledges have been steadily increasing and have become a common practice (Chan et al., 2018). However, insider equity pledges have not been fully explored, especially to controlling shareholders who control a company and make critical corporate decisions (La Porta et al., 1999). The academic literature is basically silent on how the equity pledge affects the controlling shareholder and then the company decision-making. Our paper fills this gap. In particular, our research suggests that when the controlling shareholders are faced with the margin call pressure, they have greater motivation to use their control rights to carry out M&A.

Equity pledge financing is so sought after in China’s listed companies because its market scale is vast, and more than 2/3 of the company’s shareholders have frequent pledges (Li et al., 2019). Compared with the traditional debt and equity financing methods, equity pledge has many advantages: First, it will not dilute the equity and will not cause the change of the company’s controlling shareholders. Second, compared with the direct sale of stocks, shareholders can avoid the corresponding tax burden by financing by pledging equity. At the same time, they can continue to enjoy the right to pay dividends from listed companies. Third, there are fewer approval procedures and faster access to funds. The equity pledge does not need to be approved by China Securities Regulatory Commission or the stock exchange. Still, it only requires the announcement of listed companies and the registration of pledge shareholders (holding more than 5%) in relevant institutions. Compared with other financing methods, equity pledge funds are in place faster. Fourth, the regulatory authorities have fewer regulations on the scale and use of funds, which is helpful for listed companies to supplement the sources of funds when the financing environment deteriorates. Therefore, China provides a natural experimental site for the study of controlling shareholder’s equity pledges.

A considerable number of scholars have made contributions to the controlling shareholders’ field. La Porta et al. (2000) found that listed companies in most countries globally have the characteristics of concentrated ownership, especially in countries with weak legal protection for investors. Faccio and Lang (2002) also found that the equity in some Western European countries was quite concentrated. In these countries where shares are concentrated, controlling shareholders will take advantage of their controlling position to make decisions beneficial to themselves (Faccio and Lang, 2002). In view of this, the pledge behavior of the controlling shareholder may have a material impact on the company’s activities. Also, our paper has implications for other economies.

Our motivation to study the impact of equity pledges mainly comes from the forced selling cases that caused negative publicity in the media. For example, in 2018, Yueting Jia, the controlling shareholder of LeTV, was forced to pay the margin call of its stock mortgage loan. As the stock price continues to fall, he faces the risk of control transfer and forced sale of shares. He has since been stepped down and left the company. And LetV had to be delisted after defaulting on loans. A high proportion of pledges can give the controlling shareholder enough cash, bringing significant risks after the collapse. Because of the terrible consequences of the compulsory sale of pledged shares, the controlling shareholder may have a strong incentive to use the company’s resources and try to avoid additional margin.

Although the controlling shareholders can prevent the transfer of control rights and be forced to sell their pledged shares in various ways, the company can provide price support or prevent the price from falling further by initiating mergers and acquisitions. M&A has long been regarded as an important way for enterprise merger and development (Yaghoubi et al., 2016). M&A can increase enterprise value and enable enterprises to obtain synergies (Ficery et al., 2007). In order to gain a greater competitive advantage, enterprises can also choose to acquire competitors to increase the advantages of the company (Ferrer et al., 2013). The implementation of M&A becomes a clear growth signal (Gaur et al., 2013). M&A news of listed companies as acquirers has a significant boost to their short-term stock prices (Reed et al., 2007). The reason is that, on the one hand, M&A is one of the important ways for the company to achieve exogenous growth, which will help the company to achieve the expansion of asset scale, the rapid growth of sales revenue and profits, and then bring the growth of market value to the company. On the other hand, it is difficult for small and medium-sized investors to judge the economic essence of M&A transactions of listed companies and identify potential opportunistic behaviors, such as interest transfer or hollowing out (Murray et al., 2017), and they can only respond positively to M&A news habitually or in conformity. Considering that the controlling shareholder of the pledged shares is most concerned about raising the stock price to avoid the potential transfer of control caused by margin calls and forced sale of shares, we suggest that the equity pledge of the controlling shareholder may encourage enterprises to seek value-added investment opportunities and actively participate in M&A activities. Therefore, the influence of controlling shareholders’ equity pledges on M&A decisions is an empirical problem worth studying.

This paper takes Chinese A-share listed companies as samples from 2007 to 2020 and investigates the influence of controlling shareholders’ equity pledges on M&A decisions. Our results indicate that under the pressure of margin calls, companies are more likely to conduct mergers and acquisitions when the controlling shareholders are forced to sell their shares. Moreover, when faced with great pressure, the controlling shareholder’s motivation to use M&A to raise the stock price is stronger. The results of our study have significant statistical and economic significance. In addition, to ensure the robustness of the results, we conducted additional tests to eliminate potential endogenous problems. First, we used the matching method to match the treat and control groups, and then logistic regression was performed on the samples after PSM. Our results are unaltered. Second, we used an exogenous policy in 2018 as a shock to test our results. The results by Difference In Difference (DID) still prove that our results are robust.

Our paper contributes to at least three aspects of literature. First, we link the personal behavior of insiders with that of the company. Our research contributes to this paper by adding that the personal share pledge of the controlling shareholder will affect the company’s M&A decision. Second, as far as we know, we are the first to quantify the margin call pressure of pledge in the Chinese market. We use the common standards^1^ in the market to calculate the warning line. The more equity pledges that exceed the warning line, the more pressure the controlling shareholders will face. Third, we studied the influence of controlling shareholders on M&A decision-making under pressure pledge and without pressure.

The rest of the paper framework is as follows. Section 2 reviews the literature and makes the assumptions. Section 3 describes the data and empirical approaches. Section 4 presents the main results of our research. Section 5 mainly discusses the endogeneity of our model and Section 6 concludes.

## Literature Review and Hypothesis Development

More and more literature has paid attention to the consequences of stock pledge from companies’ perspective in recent years. Chan et al. (2018) and Ouyang et al. (2019) believe that the equity pledge of insiders is related to the agency problem. They believe that companies with controlling shareholders are more likely to expropriate at the expense of minority shareholders, bringing additional shareholder risks and reducing the company’s value. Previous studies have provided empirical support for this view (Anderson and Puleo, 2020). In contrast, Li et al. (2019) argue that the threat of margin calls prevents controlling shareholders from doing bad things. Therefore, equity pledges are beneficial to increase the value of the company.

In addition, some scholars study the relationship between equity pledges and financial reporting quality. These studies show that pledge companies have the motivation to exaggerate accrual-based earnings and stabilize stock prices to avoid the pressure of additional margin (Deren and Ke, 2018). However, discretionary accruals cannot always produce positive results and eventually need to be reversed (Asija et al., 2014). In other words, share pledge companies may attract more attention from different parties, so earnings management behavior may be restricted and easy to identify (Xu et al., 2020).

Equity pledges also aggravate the separation between control rights and cash flow rights of controlling shareholders, resulting in a more serious second kind of agency problem. Therefore, the controlling shareholders with equity pledges will use their control rights to seek interests for themselves, which will endanger the interests of minority shareholders (Xiao et al., 2020). Cai (2019) showed that the pledge of major shareholders’ equity was negatively correlated with corporate performance. Chen et al. (2007) found that equity pledges by controlling shareholders increase agency costs and moral hazard issues, including earnings management, direct stock price manipulation, and venture capital project selection. Wang and Chou (2018) believe that after regulatory changes reduce the supply and incentive of equity pledges, the stock returns of companies with director pledges will increase significantly.

M&A is one of the most important ways for enterprises to develop and grow (Trichterborn et al., 2016). M&A activities will send various signals to the capital market and affect the market’s re-evaluation of the potential market value of the target enterprise. Bradley (1980) through empirical research shows that even if the company’s acquisition activity is not successful in the end, the target company’s stock will be revalued in the acquisition activity. Given this effect, Bradley et al. (1983) think that the information of M&A will revalue the listed companies and that the information of tender offer or acquisition negotiation will be passed to the managers of the target enterprises, and encourage them to adopt more effective business strategies and management activities to avoid the fate of being acquired. M&A can obtain operational synergies, which can increase corporate value, stabilize stock prices, and reduce the pledge risk of controlling shareholders. Generally, mergers and acquisitions can achieve economies of scale and scope (Devos et al., 2009) and reduce distribution costs (Alptekinoğlu and Tang, 2005). As a result, their operating efficiency and performance are improved (Healy et al., 1992). Synergies generated through acquisitions also provide a steady stream of motivation for enterprises (Ferrer et al., 2013). Since stock prices always fluctuate around the real company’s value, the M&A can stimulate the stock price, and a certain degree of mortgage can solve people’s concerns about the decline of stock price. Therefore, for enterprises with equity pledges by controlling shareholders, M&A can not only restrain the negative impact of controlling shareholders’ equity pledges but also increase the value of listed companies. As a result, controlling shareholders with equity pledges may prefer M&A to alleviate margin call pressure.

Furthermore, signals are an important channel for disseminating information to the market and reducing information asymmetry (Connelly et al., 2011), especially in the regulatory environment with a high information asymmetry and low transparency (Huang et al., 2019). We believe that the share pledge by major shareholders provides a strong signal of their personal confidence in the company’s prospects. Suppose a controlling shareholder uses stock as collateral to obtain financing. In this case, this represents a favorable assessment of the company’s future valuation relative to its current valuation, supported by private information from the controlling shareholder. Controlling shareholders’ confidence in the future sustainability of the company’s share price provides a positive signal to the market and is expected to add value to the company. In addition, investors generally pay more attention to fast-growing companies (DeGennaro, 2010) and respond positively to Acquisition announcements in China (Yang et al., 2019). M&A also serves as a signal sent by enterprises to investors. Investors seem to be aware of this behavior and underestimate the potential benefits of M&A under this pressure. Therefore, M&A is not an abuse of capital, but an important means for enterprises to explore opportunities, enhance market competitiveness and predict strong growth in the future. Thus, as investors generally have a positive attitude toward mergers and acquisitions, pledge companies may be intent on initiating mergers and acquisitions to signal growth opportunities and future value improvement. Therefore, we make a hypothesis:

*H1*: Equity pledge of controlling shareholders positively affects M&A.

Although equity pledge is a good strategy for controlling shareholder financing, if the proportion of equity pledge is too high, the controlling shareholder may lose control. In a typical stock pledge agreement, the lender specifies a maintenance condition that the market value of the pledged stock cannot decline. If the stock price falls sharply and the maintenance condition is not met the loan defaults. When this happens, the lender can sell the pledged shares. The sale of pledged shares will put more price pressure on the stock. Controlling shareholders who fail to meet margin requirements will lose their shares and control. If the market price falls to a level that violates loan maintenance requirements, the controlling shareholder can use all its powers to support the share price. Therefore, we believe that when controlling shareholders pledge a significant number of shares, they face greater margin call pressure, and they are more concerned about maintaining margin satisfaction, so they are more likely to seek mergers and acquisitions to support the share price. If share prices fall and margin calls increase, the pledge–merger relationship becomes stronger.

M&A is subtler and less risky during the pledge period. The controlling shareholder has ample incentive to issue a merger plan to prevent the share price from falling. However, the existing literature fails to mention how equity pledge affects controlling shareholders’ behavior under the pressure of margin calls, thus involving the M&A of companies. This paper fills this gap by studying the influence of equity pledge on M&A. Based on this, this paper makes the following assumption:

*H2*: As margin call pressure increases, the relationship between pledge and M&A becomes stronger.

## Data and Empirical Approach

### Data Sources

Our sample includes all Chinese A-share listed companies from 2007 to 2020. We obtained data from China Stock Market and Accounting Research (CSMAR) and WIND financial databases. These two databases are the most commonly used databases to study listed companies in China. The data of equity pledge are mainly from the WIND database, cross-verified with the iFinD^2^ database, and finally, the biased samples are removed. M&A data and control variables are from the CSMAR database. Since the main body of our study is the controlling shareholder,^3^ we excluded the sample of listed companies without controlling shareholders. Since a company may have multiple shareholders pledging at the same time in a year, we only keep the sample of controlling shareholders pledging. The reason we do this is to ensure that they have enough ownership and care about their control rights. To eliminate the concern for outliers, we winsorize all contiguous variables at 1 and 99% levels.

### Model Specification

We use the following logistic regression model to study how the equity pledge of controlling shareholders affects the decision-making of M&A:


(1) $$\begin{array}{c} M\&A_{i,t}=\beta_{0}+\beta_{1}Pledge_{i,t}/Pledge\_ratio_{i,t}+\\ \gamma Controls+Industry\;fixed\;effect+\\ Year\;fixed\;effect+\varepsilon_{i,t}\; \end{array}$$

Among them, the dependent variable M&A is a dummy variable. One is a company i that has M&A transactions in year t, otherwise 0. Pledge is a dummy variable that equals 1 if the controlling shareholder of the firm i have equity pledged in year t. According to previous studies (Chan et al., 2018; Li and Li, 2022), We control for profitability (ROA), age of listed company (Age), firm size (Size), leverage ratio (Leverage), cash ratio (Cash), Shareholding ratio of controlling shareholder (Holding_ratio), price to book value (PB), and free cash flow (FCF). The model also includes year fixed effect and industry fixed effect. In addition, the industry fixed effect in the two models is controlled by a two-digit industry classification code published by China Securities Regulatory Commission (CSRC). A more detailed explanation of variables is presented in Appendix A.

In order to study hypothesis 2, we add margin call pressure and its interaction term into Eq. (1). In the following model, we are concerned with the coefficient of *β_1_*. The margin call pressure is expressed by the proportion of the stock price below the warning line. During the equity pledge period, the more the stock price falls below the warning line, the greater the margin call pressure of controlling shareholders. In Eq. (2), we also control the industry fixed effect and year fixed effect.


(2) $$\begin{array}{c} {\mathrm{M\&A}}_{i,t}=\beta_{0}+\beta_{1}{\mathrm{Pledge}}_{i,t}*\mathrm{Margin call pressure}+\\ \beta_{2}{\mathrm{Pledge}}_{i,t}+\beta_{3}\mathrm{Margin call pressure}+\\ \gamma \mathrm{Controls}+\mathrm{Industry fixed effect}+\\ \mathrm{Year fixed effect}+\varepsilon_{i,t} \end{array}$$

### Summary Statistics

Table 1 gives basic descriptive statistics of the main variables used in our analysis. Statistics show that 25% of listed companies have controlling shareholder pledges. In the entire sample of pledges, 73% of listed companies had mergers and acquisitions in that year. The pledge of the controlling shareholder accounts for 18.18% of the shareholding ratio on average. The average age of listed companies in the sample is 10.89 years old, which is in line with the development stage of listed companies. The average margin call pressure is close to zero, meaning that about half of the controlling shareholders did not experience a fall into the warning zone, most likely because they made acquisitions immediately after the pledge to boost the stock price.

**Table 1:** Descriptive statistics.

VarName	Obs	Mean	*SD*	Min	Median	Max
M&A	29,333	0.73	0.441	0	1	1
Pledge	29,333	0.25	0.433	0	0	1
Pledge_ratio	29,333	18.18	32.923	0	0	100
Age	29,333	10.89	7.225	1	10	31
ROA	29,333	0.03	0.560	−51.94684	0.036895	22.00512
Leverage	29,333	0.47	1.637	0.00708	0.431062	178.3455
Cash	29,333	1.01	2.766	−4.358657	0.379802	167.544
Size (std)	29,333	0.00	1.000	−0.238084	−0.1696242	36.16212
Shareholding_ratio	29,333	36.91	15.235	0.1643	35.48	89.99
PB	29,333	4.83	25.037	0	2.785926	2000.87
FCF (std)	29,333	0.00	1.000	−25.31188	−0.0250361	44.84508
Margin_call_pressure	7,145	−0.06	0.423	−0.7941454	−0.1129896	0.895261

Table 2 reports the univariate differences between the pledge and non-pledge groups of controlling shareholders. In the whole sample of pledges, the probability of M&A of controlling shareholders is significantly higher than that of non-controlling shareholders. This is a preliminary test of our hypothesis. The average size of companies pledged by controlling shareholders is more significant than that of non-controlling shareholders. In addition, the FCF of controlling shareholders in the pledge sample is also more significant than that of non-controlling shareholders, which is likely due to the financing generated after the equity pledge.

**Table 2:** Test for differences between univariate groups.

Variables	Non-controlling shareholder pledge		Controlling shareholder pledge		MeanDiff	*T*-value
	Obs	Mean	Obs	Mean		
M&A	22,005	0.692	7,328	0.863	−0.171	−33.715^***^
Pledge_ratio	22,005	2.844	7,328	64.230	−61.387	−172.162^***^
Age	22,005	11.036	7,328	10.457	0.579	6.181^***^
ROA	22,005	0.027	7,328	0.025	0.002	0.505
Leverage	22,005	0.485	7,328	0.442	0.043	3.356^***^
Cash	22,005	1.118	7,328	0.672	0.445	16.932^***^
Size (std)	22,005	0.018	7,328	−0.054	0.071	7.919^***^
Shareholding_ratio	22,005	37.853	7,328	34.091	3.762	19.729^***^
PB	22,005	4.937	7,328	4.500	0.437	1.719^*^
FCF (std)	22,005	0.010	7,328	−0.031	0.041	3.463^***^

*** *p* < 0.01;

^**^p < 0.05;

* *p* < 0.1.

Table 3 is the correlation coefficient matrix between variables. It can be concluded that basically all variable relations are less than 0.5, so there is no need to worry too much about possible multicollinearity.

**Table 3:** Pairwise correlations.

Variables	(1)	(2)	(3)	(4)	(5)	(6)	(7)	(8)	(9)	(10)
(1) M&A	1.000									
(2) Pledge	0.168^***^	1.000								
(3) ROA	−0.012^**^	−0.002	1.000							
(4) Age	0.117^***^	−0.035^***^	−0.036^***^	1.000						
(5) Leverage	0.016^***^	−0.011^**^	−0.678^***^	0.064^***^	1.000					
(6) Cash	−0.104^***^	−0.070^***^	0.022^***^	−0.187^***^	−0.058^***^	1.000				
(7) Size (std)	0.021^***^	−0.031^***^	0.007	0.072^***^	0.013^**^	−0.043^***^	1.000			
(8) PB	0.009	−0.008	0.059^***^	0.023^***^	0.005	0.013^**^	−0.017^***^	1.000		
(9) FCF (std)	−0.007	−0.018^***^	0.011^*^	0.027^***^	−0.002	−0.015^**^	0.183^***^	−0.004	1.000	
(10) Shareholding_ratio	−0.130^***^	−0.107^***^	0.031^***^	−0.156^***^	−0.009	0.026^***^	0.149^***^	−0.043^***^	0.054^***^	1.000

*** *p* < 0.01;

** *p* < 0.05;

* *p* < 0.1.

## Empirical Results

### Controlling Shareholders Pledge and M&A

Firstly, this paper makes an empirical analysis on the influence of controlling shareholder’s equity pledges on M&A decisions. Logistic regression is used to run our baseline model of Eq. (1). The coefficients of logistic regression reported by column (1) and column (3), column (2), and column (4) are their average bias effect, respectively, for interpretation. In column (1), the coefficient of Pledge is 0.956, which means that pledge by controlling shareholders plays a positive role in M&A, and it was statistically significant at the 1% level. For the sake of explanation, we present the average partial effect in column (2). The results show that the probability of pledged M&A is 17.3% higher than that of non-pledged M&A. In order to explain the impact of pledge on M&A in more detail, we use pledge_ratio instead of the pledge. Column (3) and column (4) are also positive and significant in the economic sense. The results show that each unit increase in pledge rate increases the probability of M&A by 0.2%.

Taken together, these results indicate that controlling shareholders’ equity pledge is more correlated with M&A, supporting controlling shareholders’ strong motivation to prevent control transfer through M&A (Table 4).

**Table 4:** Logistic regression and average partial effect.

	(1)	(2)	(3)	(4)
Variables	Logistic	APE	Logistic	APE
Pledge	0.956^***^	0.173^***^		
	(0.0405)	(0.00716)		
Pledge_ratio			0.0150^***^	0.00272^***^
			(0.000634)	(0.000111)
ROA	−0.00498	−0.000901	0.00372	0.000671
	(0.0495)	(0.00895)	(0.0465)	(0.00839)
Age	0.0244^***^	0.00442^***^	0.0223^***^	0.00403^***^
	(0.00231)	(0.000414)	(0.00229)	(0.000411)
Leverage	0.00831	0.00150	0.00971	0.00175
	(0.0212)	(0.00383)	(0.0219)	(0.00395)
Cash	−0.0553^***^	−0.01000^***^	−0.0551^***^	−0.00995^***^
	(0.0101)	(0.00181)	(0.0101)	(0.00181)
Size (std)	0.0845^***^	0.0153^***^	0.0867^***^	0.0157^***^
	(0.0237)	(0.00429)	(0.0244)	(0.00441)
PB	0.000567	0.000103	0.000589	0.000106
	(0.000888)	(0.000161)	(0.000902)	(0.000163)
FCF (std)	−0.0168	−0.00304	−0.0173	−0.00312
	(0.0170)	(0.00308)	(0.0172)	(0.00311)
Shareholding_ratio	−0.0155^***^	−0.00280^***^	−0.0140^***^	−0.00252^***^
	(0.000934)	(0.000167)	(0.000943)	(0.000168)
Constant	1.338^***^		1.309^***^	
	(0.143)		(0.144)	
Year fixed effect	Yes	Yes	Yes	Yes
Industry fixed effect	Yes	Yes	Yes	Yes
Observations	29,333	29,333	29,333	29,333

Robust SEs in parentheses.

*** *p* < 0.01;

^**^p < 0.05;

^*^p < 0.1.

### Margin Call Pressure, Pledge, and M&A

Margin call pressure is a massive problem in the research of pledges and M&A. We use a common standard in the market, namely, pledge rate (40%), margin line (130%), and warning line (150%) to calculate margin call pressure. When the stock price is lower than the warning line price, the controlling shareholder faces greater pressure of margin payment, and when it is close to the close position, it may be forced to sell shares and lose control. We can draw a conclusion from the coefficient of interaction terms. Table 5 proves our hypothesis that the greater the margin call pressure faced by the controlling shareholder, the higher the possibility of using M&A to raise the stock price.

**Table 5:** Margin call pressure, pledge, and M&A.

	(1)
Variables	Logistic
Pledge_ratio	0.0105^***^
	(0.00135)
Margin	−0.542^***^
	(0.176)
Pledge_ratio ^*^ margin	0.00864^***^
	(0.00280)
ROA	−0.102
	(0.614)
Age	−0.0293^***^
	(0.00668)
Leverage	0.836^***^
	(0.279)
Cash	−0.0271
	(0.0275)
Size (std)	0.799^**^
	(0.358)
PB	0.0187^*^
	(0.0110)
FCF (std)	0.116
	(0.119)
Shareholding_ratio	−0.0187^***^
	(0.00276)
Constant	1.611^***^
	(0.368)
Observations	7,141

Robust standard errors in parentheses.

*** *p* < 0.01;

** *p* < 0.05;

* *p* < 0.1.

## Addressing Endogeneity Concerns

### Propensity Score Matching

Since we are not clear about the use of funds after the equity pledge, one might suspect that there is a sample selection problem. That is, the funds pledged may be used for M&A, or for other purposes. To solve this problem, we used propensity score matching (PSM) to alleviate the endogenous problems. This method can create corresponding research conditions for treat group and control group. The controlling shareholder pledge group is the treat group in this study, otherwise is the control group. We use the following covariates to match. ROA, Age, Leverage, Cash, Size (standardized), PB, FCF (standardized), and Shareholding_ratio. After the PSM process, 12,940 observations were left after 1:1 matching. Results are shown in Table 6. The coefficients are still significantly positive. In general, the controlling shareholder pledge promotes the possibility of M&A.

**Table 6:** The logistic regression after Propensity score matching (PSM).

	(1)	(2)
Variables	Logistic	APE
Pledge	0.863^***^	0.129^***^
	(0.0525)	(0.00768)
roa	0.00120	0.000179
	(0.357)	(0.0536)
Age	0.00529	0.000794
	(0.00402)	(0.000602)
Leverage	0.972^***^	0.146^***^
	(0.164)	(0.0245)
Cash	−0.0461^***^	−0.00692^***^
	(0.0174)	(0.00261)
std_Size	0.174^**^	0.0261^**^
	(0.0744)	(0.0112)
pb	−0.000404	−6.06e-05
	(0.000636)	(9.53e-05)
std_fcff	−0.0220	−0.00329
	(0.0406)	(0.00609)
Shareholding_ratio	−0.0174^***^	−0.00261^***^
	(0.00165)	(0.000246)
Constant	1.181^***^	
	(0.255)	
Observations	12,940	12,940

Robust SEs in parentheses.

*** *p* < 0.01;

** *p* < 0.05;

^*^p < 0.1.

### Difference in Difference

On March 12, 2018, the Measures for Stock Pledge Repurchase Transactions, Registration, and Settlement Business officially came into effect, standardizing the stock pledge market from the aspects of financing threshold, concentration, pledge rate, financing purposes, and so on, and putting forward stricter requirements on the business behaviors of securities brokers. In order to alleviate people’s endogenous concerns, we chose this policy as an exogenous shock. Considering the possible influence of other policies, samples of plus or minus 2 years in 2018 are selected. To capture the time-trend effect, we constructed a variable Post, where 1 represents after the implementation of the policy in 2018 and 0 represents before 2018. The key variable we are interested in is the interaction term between Treat and Post, which captures the net effect of exogenous changes in controlling shareholder equity pledge on M&A propensity (Table 7).

**Table 7:** Difference in difference (DID) analysis.

	(1)
Variables	DID
Post ^*^ Treat	0.0251^**^
	(0.0104)
roa	0.00443
	(0.00463)
Age	−0.00102
	(0.00238)
Leverage	0.00187
	(0.00176)
pb	0.000140^***^
	(4.97e-05)
std_fcff	0.00228
	(0.00381)
std_Size	0.0297^***^
	(0.00783)
Constant	0.800^***^
	(0.0280)
Firm fixed effect	Control
Year fixed effect	Control
Observations	16,479
Number of id	3,747
R-squared	0.010

Robust SEs in parentheses.

*** *p* < 0.01;

** *p* < 0.05;

* *p* < 0.1.

## Conclusion

Insider pledge of stocks is common all over the world. Although scandals are often involved in the equity pledge of controlling shareholders in the market, there is still an incomplete understanding of the economic consequences of pledges and whether and how the individual pledge behavior affects the company’s decision-making. We fill this gap by studying the influence of controlling shareholders’ share pledges on M&A. We put forward a margin call pressure hypothesis, that is, controlling shareholders can take advantage of their own advantages to initiate M&A, so as to reduce the potential margin call pressure when they pledge stocks for personal loans. Our hypothesis predicts that when the controlling shareholders have pledged a considerable part of the equity and there is great pressure to recover the deposit, they are more likely to start the M&A scheme. In order to prevent the loss of control rights, we find that when the controlling shareholder pledges a higher proportion of shares, the company is more likely to start the M&A plan. The possibility of M&A is particularly high when the company has experienced a sharp drop in stock price recently and is about to ask for additional margin. In addition, our endogenous test is also very robust.

The limitation of this study is that we mainly focus on domestic M&A and have not considered cross-border M&A. The main reason is that the sample of cross-border M&A is too small. In the future, we will continue to expand the research on this issue, including the different effects on the time series before, during, and after the equity pledge.

Generally speaking, our findings are consistent with the explanation of control rights, that is, the controlling shareholders use M&A to relieve the pressure of additional margin, and their motivation is to protect their control of company resources and gain private interests. External regulatory policies strengthen this relationship. This paper helps to deepen the understanding of investors and regulators on the economic consequences of controlling shareholders’ equity pledges. We provide an important policy basis for the governance of the share pledge. In the future, we should deepen the policy supervision of controlling shareholders’ equity pledge to prevent controlling shareholders’ self-interest behavior.

## Data Availability Statement

The raw data supporting the conclusions of this article will be made available by the authors, without undue reservation.

## Author Contributions

XL wrote the manuscript. QL provided an idea. All authors contributed to the article and approved the submitted version.

## Funding

We gratefully acknowledge the program of the Tutor Academic Leadership Program of Shanghai International Studies Universities (2020114224) and the financial support of the Humanities and Social Science Grant from the Ministry of Education, China (#20YJA630098).

## Conflict of Interest

The authors declare that the research was conducted in the absence of any commercial or financial relationships that could be construed as a potential conflict of interest.

## Publisher’s Note

All claims expressed in this article are solely those of the authors and do not necessarily represent those of their affiliated organizations, or those of the publisher, the editors and the reviewers. Any product that may be evaluated in this article, or claim that may be made by its manufacturer, is not guaranteed or endorsed by the publisher.

## Footnotes

^1^Pledge rate 40%, close position 130%, warning line 150%.

^2^iFinD financial data terminal is a software system owned by Tonghuashun Company that provides research and investment decision services for institutional customers. Its core functions include multi-dimensional information of different financial varieties, several macro industry databases, and research tools such as asset management systems and valuation models. Institutional clients include securities companies, fund companies, banks, futures, investment consulting, media, universities, governments, listed companies, etc.

^3^Controlling shareholders are defined as those who have more than 50% of the voting rights or who have the right to make decisions.

## Appendix

**Appendix A:** Variables definition.

Variable	Definition
M&A	Dependent variable. 1 indicates that the controlling shareholder has made an M&A, otherwise 0
Pledge	Independent variable. 1 indicates that the controlling shareholder has equity pledged, otherwise it is 0
Pledge_ratio	Proportion of controlling shareholder pledge to shareholding
Margin call pressure	Warning line price minus the lowest price of the shares during the pledge period divided by the warning line price
Warning line price	The stock cost at the time of pledge (that is, the stock price at the time of pledge) ^*^ the pledge rate ^*^ the warning line ratio. (According to the standard, the pledge rate is 40%, the warning line is 150%, and the closing line is 130%.)
FCF (std)	Standardized free cash flow. Source: CSMAR
Age	Age of listed companies. Source: CSMAR
Leverage	Firm financial leverage, calculated by total long-term debts divided by total assets. Source: CSMAR
Cash	The cash ratio is a measurement of a company’s liquidity, specifically the ratio of a company’s total cash and cash equivalents to its current liabilities. Source: CSMAR
ROA	Return on Assets. Source: CSMAR
Size (std)	Standardized book value. Source: CSMAR
pb	Price to book value. Source: CSMAR
Shareholding ratio	Shareholding ratio of controlling shareholder. Source: CSMAR
